# Targeting elongation factor 2 in *Anopheles gambiae*: molecular insights and implications for malaria vector control

**DOI:** 10.3389/finsc.2026.1869625

**Published:** 2026-08-13

**Authors:** Adebanke E. Ogundipe, Fabrice B. Kernyuy, Titilope M. Dokunmu, Emeka E. Iweala, Olubanke O. Ogunlana

**Affiliations:** 1 Department of Biochemistry, Covenant University, Ota, Nigeria; 2 Covenant Applied Informatics and Communication Africa Centre of Excellence (CApIC-ACE), Ota, Nigeria

**Keywords:** *Anopheles gambiae*, elongation factor 2, insecticide resistance, malaria, vector control

## Abstract

Malaria remains one of the most devastating infectious diseases globally, with *Anopheles gambiae* serving as the principal vector across sub-Saharan Africa. The effectiveness of traditional vector control methods is currently under threat due to the rapid rise of pesticide resistance, which calls for the discovery of new molecular targets. The GTP-dependent GTPase known as Eukaryotic Elongation Factor 2 (EF-2; encoded by AGAP009441 in *An. gambiae*) is essential for cellular viability because it catalyzes the translocation stage of ribosomal protein production. EF-2 was identified as an important gene in An. gambiae by machine learning-based computational screening, and RNA interference (RNAi)-mediated knockdown experimentally verified that EF-2 silencing dramatically shortens mosquito longevity (p < 0.0001). Despite the fact that EF-2 and its human homolog share about 79% of the same protein sequence, species-specific structural characteristics may provide opportunities for selective targeting. Additionally, a comparative dN/dS analysis was carried out on EF-2 across *Anopheles* species using the HyPhy Datamonkey platform. The dN/dS results revealed high evolutionary conservation (ω = 0.0198) and are consistent with purifying selection, but not with positive selection. The current understanding of EF-2 biology, its validation as a vector control target, suitable intervention modalities like RNA interference and small-molecule inhibition, and the major issues of selectivity, delivery, and ecological safety that need to be resolved prior to translational application are all summarized in this review. EF-2 is a promising but understudied contender whose complete potential in integrated malaria vector control necessitates immediate and ongoing research.

## 1 Introduction

Malaria is still a major public health concern, and in the year 2024, there were an anticipated 282 million infections and 610,000 fatalities worldwide (1). Approximately 94% of cases and 95% of deaths occur in Sub-Saharan Africa, with pregnant women and children under five years old at the highest risk (1). *P. falciparum* is responsible for the great majority of severe disease and mortality in Africa (1). The predominant malaria vector in most of sub-Saharan Africa is *Anopheles gambiae sensu stricto*, which combines high anthropophily, endophily, and vectorial competence to maintain high levels of *Plasmodium falciparum* transmission (2, 3). Current vector control strategies rely primarily on long-lasting insecticidal nets (LLINs) and indoor residual spraying (IRS), predominantly using pyrethroids and, increasingly, non-pyrethroid alternatives such as piperonyl butoxide (PBO)-based formulations (4, 5). However, these interventions are now seriously threatened by the quick and widespread establishment of insecticide resistance throughout *An. gambiae* populations (6). Target-site mutations in the voltage-gated sodium channel (knockdown resistance, or kdr) and metabolic processes controlled by elevated cytochrome P450 monooxygenases, glutathione-S-transferases, and esterases are two aspects of pyrethroid resistance (7, 8). In many West and Central African settings, allele frequencies of the kdr-W (L1014F) mutation are near-fixation, rendering standard pyrethroid insecticides largely ineffective (8).

These facts highlight how urgent it is to find new molecular targets for malaria vector control. Particularly attractive possibilities are essential genes whose disruption results in the organism’s death. *An. gambiae’s* gene AGAP009441 encodes Elongation Factor 2 (EF-2), a conserved GTPase essential for ribosomal translocation during protein synthesis. Recent computational and experimental work has identified EF-2 as an essential gene in *An. gambiae* and demonstrated that its silencing via RNAi significantly reduces mosquito survival (9). This review critically evaluates the biological significance of EF-2, its candidacy as a vector control target, potential intervention modalities, –and the challenges that must be overcome for translational progress.

## 2 Biological role of EF-2 in eukaryotes

Eukaryotic Elongation Factor 2 (eEF2) is a ~95 kDa GTP-binding protein that mediates the translocation step of ribosomal translation elongation, an obligate and irreversible commitment in protein synthesis (10, 11). Following peptide bond formation, eEF2 catalyzes the coordinated movement of peptidyl-tRNA and deacylated tRNA from the A-site to the P-site and P-site to E-site of the 80S ribosome, respectively, while simultaneously translocating mRNA by one codon in a GTP hydrolysis-dependent manner (11, 12). The process clears the A-site for the incoming aminoacyl-tRNA by changing the ribosome’s shape from pre-translocational (PRE) to post-translocational (POST). The protein has six structural domains (I-V and G). Domain IV, which contains the unique post-translationally modified histidine residue known as diphthamide, is crucial for maintaining translational integrity and ribosome decoding accuracy (13, 14).

Under conditions of energy stress, nutritional restriction, or TORC1 pathway blockage, the specialized kinase eEF2K (also known as CaMKIII) phosphorylates eEF2 at Thr-56, inactivating eEF2 and thus reducing translational elongation globally (15, 16). This regulatory circuit positions eEF2 as a major control point governing the rate of cellular protein production (16). Unlike Elongation Factor 1-alpha (eEF1A), which is encoded by multiple gene isoforms in many organisms, eEF2 is typically encoded by a single gene in eukaryotes, making it a more precise RNAi target with reduced compensation risk (9, 17).

eEF2 is broadly conserved across eukaryotes and is classified as an essential gene in multiple model organisms, including *Saccharomyces cerevisiae*, *Caenorhabditis elegans*, *Drosophila melanogaster*, and mammals (9). Its indispensability for cell viability, combined with its role in one of the highest-throughput biosynthetic processes in proliferating cells, makes it an attractive target in organisms where disruption of protein synthesis is lethal. It’s targeted by bacterial toxins (diphtheria toxin, Pseudomonas exotoxin A), which ADP-ribosylate the diphthamide residue, irreversibly inactivating EF-2 and halting protein synthesis, further demonstrating the lethal consequence of EF-2 inhibition (13).

## 3 EF-2 in *Anopheles gambiae*: gene, protein, and functional evidence

In *An. gambiae*, EF-2 is encoded by locus AGAP009441 (hereafter Elf2). According to the findings of the sequence analysis, it has been shown that Elf2 is around 78.7% similar to the eEF2 gene in humans at the protein level (E-value = 0.0), and 79.5% similar to EEF2 at the genetic level (E-value = 2.5 × 10⁻^94^). Although high conservation implies functional orthology and biological significance of the protein, it raises issues of the drug targeting selectivity (9).

Transcriptomic profiling across *An. gambiae* life stages reveals that Elf2 is highly expressed, with transcripts per million (TPM) values substantially above background in available RNA-seq datasets, consistent with the constitutive and high-throughput demand for translational elongation in metabolically active adult mosquitoes (9). This stands in contrast to Elongation Factor 1-alpha (encoded by AGAP007406), which has an isoform (AGAP003541) that can compensate upon silencing of the primary isoform, a compensation mechanism absent in the single-copy Elf2 gene, thereby increasing its reliability as an RNAi target (9).

The essentiality of Elf2 in *An. gambiae* was computationally predicted using the CLEARER (CLassifier of Essentiality AcRoss EukaRyote) machine learning algorithm, which was trained across six model eukaryotic organisms and predicted 8.2% of *An. gambiae* genes (852 of 10,426) as essential under both cellular and organism-level criteria (9). Elf2 was among these doubly-essential predictions. Notably, Elf2 was ranked among the top-expressed non-ribosomal essential genes in the transcriptomic data, reflecting its high constitutive activity and physiological centrality in adult female mosquitoes (9).

## 4 EF-2 as a target for vector control

### 4.1 EF-2 comparative evolutionary conservation

The degree of large-sequence conservation in EF-2 across *Anopheles* species indicates that this protein could be the key to developing management strategies against malaria vectors. In order to add more evidence, a short codon-based dN/dS study was performed using HyPhy Datamonkey web tool for the codon sequences of EF-2 from the main *Anopheles species* (18). The analysis in **Table 1** shows that the value of dN/dS was very low (ω=0.0198) suggesting that EF-2 is subjected to strong purifying selection. Also, codons showed that there were 221 codons under evident and strong purifying selection, while there were no codons undergoing positive selection. Moreover, codon-level analysis has revealed a total of 221 positions that exhibit significant recurrent purifying selection while no codons exhibited any signs of recurrent positive selection. These observations imply that EF-2 has preserved itself during evolution of the main malaria vectors due to severe constraints involved in the processes of protein synthesis. This aspect of evolutionary stability makes EF-2 very attractive as a promising target when developing new wide-spectrum vector control strategies.

**Table 1:** Summary of EF-2 comparative evolutionary conservation.

Parameter	Findings
Five (5) *Anopheles* species analyzed	*An. gambiae, An. arabiensis, An. stephensi, An. funestus, An. minimus*
dN/dS (w) ratio	0.0198
Number of codon analyzed	844
Number of negative selection	221
Number of positive selection	0
Significance threshold	p ≤ 0.10
Evolutionary inference	Strong purifying selection

### 4.2 RNA interference approaches

The most direct experimental evidence for EF-2 as a vector control target comes from RNAi knockdown studies in *An. gambiae* G3 colony mosquitoes. Adedeji et al. (2024) designed a double-stranded RNA (dsRNA) targeting AGAP009441 and delivered it via intrathoracic microinjection into adult female mosquitoes. dsRNA injection achieved a knockdown efficiency of 61%, and Elf2-silenced mosquitoes exhibited significantly reduced survival compared to all control groups (non-injected, PBS-injected, and LacZ-injected; all comparisons p < 0.0001). The findings of this phenotype indicated that to limit the expression of Elf2 partially is enough for the mosquito to become less fit, which means that it should be considered characterized as an essential gene at organism level (9). Many studies concerned with various processes in the body (e.g., fecundity, immunity, insecticide resistance) have proven that RNA interference (RNAi) can be used in An. gambiae vector control (19, 20). Micro-injection is the most commonly used method of RNAi delivery in the laboratory, still there are various systems under development for applied purposes, such as consuming feed with dsRNA expressed by bacterial vectors, feed made of encapsulated chitosan nanoparticles, yeast-based delivery systems, and engineered gut symbiotic bacteria (21, 22). Yeast-based delivery is the most effective in terms of larval mortality in mosquitoes (22) while symbiont-mediated delivery conducted with the use of improved *Serratia fonticola* bacteria seems promising for the larvae management in *An. stephensi* (21). All these possibilities can be used for EF-2-directed RNAi process application on large scale.

### 4.3 Small-molecule inhibitors and natural compounds

EF-2 is recognized as a target for drug development in many different species, including using RNA interference. Sordarin, a natural product derived from Monosporium apiospermum, acts as an inhibitor of fungal eEF2 by binding to the ribosome and preventing dissociation of the protein after GTP hydrolysis. Importantly, sordarin has antifungal activity that is specific to certain species since it uses the structural differences of eEF2 in mammals and fungi (23, 24). While sordarin has been tested extensively in fungi, its potential to inhibit mosquito EF-2 has yet to be fully investigated. Additionally, sordarin has not translated to an insecticide due to the issue of compound optimization, bioavailability. delivery and detailed toxicological assessment. This precedent demonstrates that small-molecule selectivity targeting EF-2 across species boundaries is structurally achievable. In the malaria parasite context, computational modeling identified *P. falciparum* EF-2 as a key candidate for transmission-blocking interventions (25) further highlighting the broader anti-parasitic relevance of EF-2 inhibition within the malaria life cycle.

For *An. gambiae* Elf2 specifically, the preconditions for *in silico* inhibitor discovery are satisfied: the protein sequence is defined, the human ortholog crystal structure (PDB: multiple depositions) provides a structural template for comparative modeling, and species-specific sequence divergences (~21% difference from the human protein) offer potential selectivity pockets (9). An analogous selective-inhibitor strategy has been demonstrated for *An. gambiae* acetylcholinesterase, where an unpaired cysteine residue present in the mosquito but absent in humans was successfully exploited to design selective inhibitors (9). Identifying equivalent species-specific features in Elf2 through comparative structural genomics represents a high-priority research avenue. Diamide insecticide is another example as they selectively target even though homologous receptors exist in mammals. Also, the selective activity of the neonicotinoid and spinosyn insecticides targeting insect nicotinic acetylcholine receptors (nAChRs) proves that a selective activity is possible with different receptor subunit composition and the ligand-binding site between insects and vertebrates. In comparison, with targets such as voltage gated sodium channel, which are well-conserved, insect and vertebrate proteins are likely more similar in terms of their structures, which could potentially increase the off-target effect. All the examples stated highlighted that having a human orthologue only does not conclude a protein as a suitable insecticidal target (9).

### 4.4 Potential advantages of EF-2 as a novel molecular target

EF-2, a novel molecular target, works through mechanisms that is different from the known targets, such as sodium channels, acetylcholinesterase, or GABA receptors (7, 26). Consequently, the approaches targeting EF-2 are promising in terms of reducing the chances of cross-resistance. However, it needs to be tested experimentally, considering that mosquitoes might have different genetic, metabolic, and physiological mechanisms of resistance.

Secondly, in *An. gambiae*, EF-2 is encoded by a single gene without a viable isoform backup, which lowers the likelihood of target-mediated resistance through gene redundancy (9). Alternative splicing and the production of isoforms under stressful conditions cannot be ruled out, as EF-2 is a multi-exon gene. Therefore, transcriptomics and functional studies under different physiological stress conditions should be further investigated to determine whether different EF-2 isoforms are present and whether the suggested decrease in target resistance occurs.

Thirdly, EF-2 is crucial for protein synthesis, making it a promising target for vector control research. However, even though this is true, it’s worth noting that this is not specific for EF-2, as the known targets of insecticides (like voltage-gated sodium channels, acetylcholinesterase, and GABA receptors) also have vital biological functions in mosquitoes. Thus, the significance of EF-2 does not stem solely from its essentiality, but rather from whether future studies will show that targeting EF-2 can resolve the issues faced by current insecticide targets, such as resistance, without losing efficacy and specificity (27).

## 5 Challenges and limitations

### 5.1 Target specificity and selectivity

**Figure 1:**
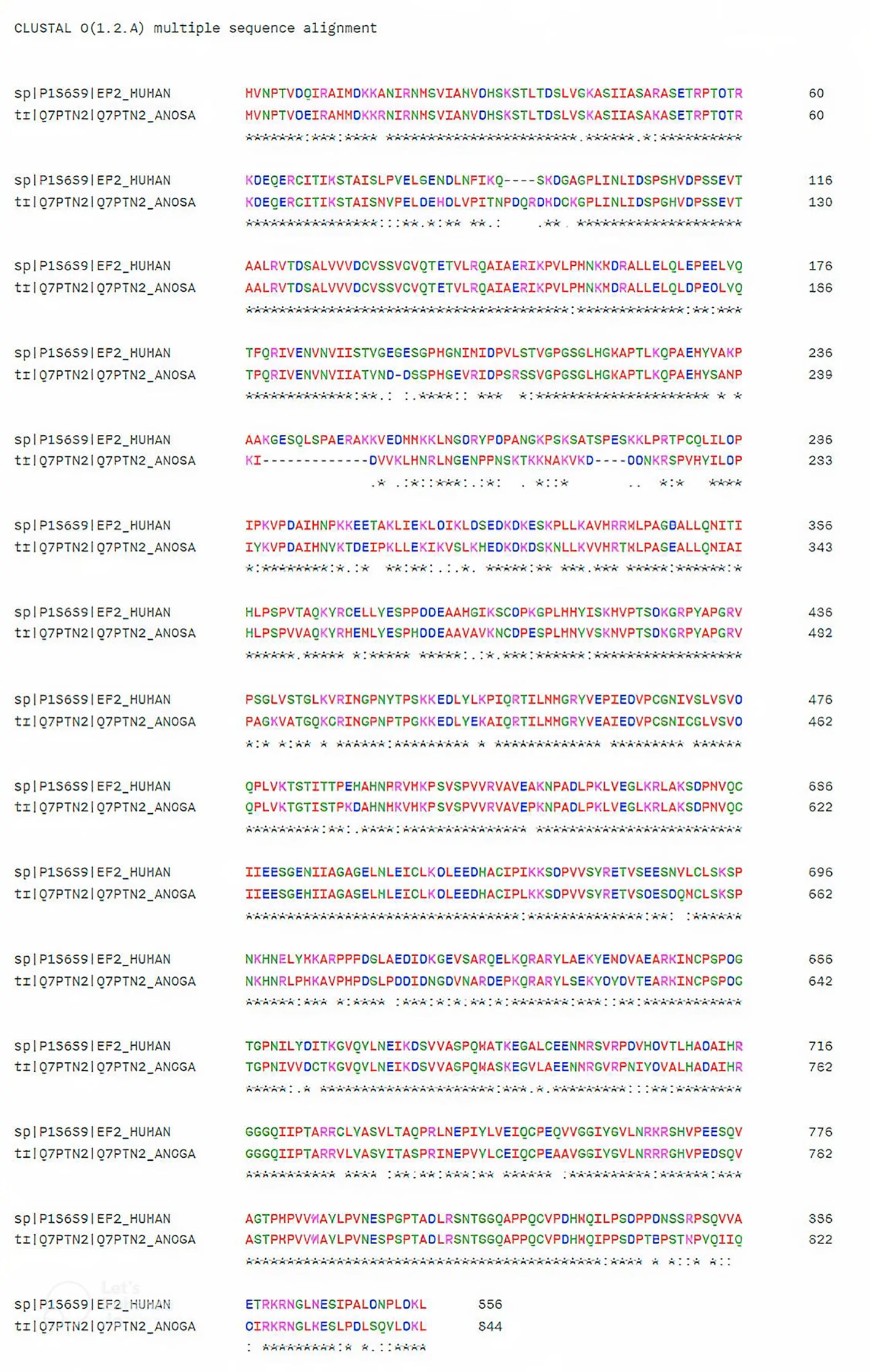
Sequence alignment of human EF2 and *Anopheles gambiae* EF2 proteins. Multiple sequence alignment using Clustal Omega showing comparative amino acid sequences. Asterisks (*) denote identical residues; colons (): indicate conserved substitutions; dots (.) represent semi-conserved changes. The alignment spans 858 amino acids (human) and 844 amino acids *(An. gambiae)*, with overall identity of 80.71%. Regions of divergence highlight potential targets for selective inhibitor design and RNAi specificity.

The strong sequence identity (80.71% overall) with the human EEF2 orthologue presents the biggest obstacle for both RNA interference and small-molecule methods targeting Elf2. A sequence alignment of human EF2 (UniProt: P13639) and *Anopheles gambiae* EF2 (UniProt: Q7PTN2) reveals conserved functional domains interspersed with regions of divergence that may be exploitable for selective targeting (**Figure 1**). The similarity between EF-2 in mosquitoes and EF-2 in humans is confirmed by the high degree of conservation at the DNA and protein level. While the majority of the amino acid sequences of EF-2 from various species exhibit great homology; this does not necessarily imply identical kinetic properties (e.g., inhibition) for inhibitors that are selected against each EF-2. In particular, minor variations in the amino acid sequence of a protein can influence its conformation in space and/or on its surface, as well as its electrostatic interactions and the architecture of the ligand binding pocket, all of which have a direct effect on either the affinity or specificity of binding. This necessitates careful structure-activity relationship (SAR) research on small-molecule inhibitors to determine or engineer selectivity, since substances with sufficient affinity for mosquito Elf2 may also inhibit human EEF2 and pose a toxicological concern. Therefore, detailed comparative structural analysis of *Anopheles gambiae* EF-2 and human EEF2 may reveal exploitable molecular differences that support selective mosquito targeting while minimizing off-target effects in humans (9).

An inhibitory effect on human EEF2 may present a significant safety risk due to the importance of EEF2 in protein synthesis, cellular growth and maintaining homeostasis in human organs. Inhibition of human EEF2 in a manner that is non-selective is likely to affect the process of translational elongation which could then deteriorate how the cell is functioning normally and especially when in a tissue that has a high metabolic rate. The fact that EEF2 has been shown to be rendered an irreversibly inactive enzyme by toxins such as diphtheria toxin and Pseudomonas exotoxin A confirms that this is indeed a concern. However, if sufficiently different species-specific structure exists, it may still be possible to selectively exploit conserved targets; therefore, EF-2 mosquito control without harming human EEF2 will require a detailed structural comparison analysis, a selectivity assay, and a toxicology evaluation. Sequence specificity for RNAi is intrinsic (dsRNA sequences are made to match the mRNA of the target species), but prior to field deployment, thorough bioinformatic off-target analysis is necessary to prevent cross-reactivity with non-target organisms that share homologous sequences, like beneficial insects or aquatic invertebrates (20).

### 5.2 Delivery systems and field application

There are major logistical obstacles to converting RNA interference from the laboratory to practical vector control. Even though many platforms for the delivery of RNAi have been invented, the transition from research to the use of such technologies for combating malaria vectors still stands as a serious challenge. Some of the difficulties include the ability to produce double-stranded RNA (dsRNA) in cost-effective ways on a large scale, as well as keeping its stability in environmental conditions, ensuring the effective absorption of dsRNA by the wild mosquito populations, complying with the regulations and lessening the danger to non-target species (28–30). That is why large-scale semi-field and field studies must precede the application of the EF-2 targeting RNAi solutions in the mosquito control strategy.

### 5.3 Resistance risks

Targeted resistance is not eliminated despite the fact that EF-2’s critical function and single-copy state lessen some resistance pathways. It is possible for mosquito populations under selection pressure from EF-2-directed interventions to develop resistance through point mutations in the dsRNA target region, modifications to dsRNA absorption routes, overexpression of competing translational factors, or decreased systemic RNAi spread (20, 31). Resistance to RNAi has been documented in other insects, particularly when dsRNA concentrations fall below threshold levels. Resistance management strategies, including rotating target genes, deploying dsRNA targeting multiple non-overlapping gene sequences, or combining RNAi with other interventions, must be incorporated into any deployment framework from the outset (32).

Besides the established mechanisms of resistance, alternative splicing might serve as a potential adaptive response to selective pressure. Currently, there is no evidence of EF-2 isoforms under stress in *An. gambiae*. However, the emergence of alternative isoforms with a modified shape, location, or expression may potentially decrease the vulnerability of *An. gambiae* to EF-2 focused treatments. This possibility should be further studied in transcriptomic and functional terms to establish if alternative splicing takes part in the development of resistance (33).

## 6 Environmental and ecological considerations

A thorough evaluation of the ecological effects of using EF-2-targeted compounds in malaria-endemic areas is necessary. Although sequence-based RNAi selectivity is a significant benefit, dsRNA molecules released into aquatic larval environments may affect non-target invertebrates with similar sequences. Globally, dsRNA-based biopesticide regulatory frameworks are still being developed (29), and long-term ecological effects of population suppression of *An. gambiae*, an abundant species in many African ecosystems, the role of this species in biodiversity and food webs remains incompletely understood. Baseline ecological risk assessments must be integrated into any translational pipeline.

## 7 Future perspectives

Several critical research gaps must be addressed to advance EF-2 as an operational vector control target. First, comprehensive structural characterization of *An. gambiae* EF-2, including high-resolution crystal structures or cryo-EM models, is needed to map species-specific surface features amenable to selective small-molecule binding. Comparative structural genomics across mosquito and human EF-2 orthologues, informed by approaches successfully applied to other *An. gambiae* proteins (e.g., acetylcholinesterase), should be prioritized (9). Second, the transcriptomic and proteomic profiles of EF-2 across all life stages, larvae, pupae, adult males, and blood-fed females, require detailed characterization to identify optimal intervention windows, as current data are primarily derived from adult females (9).

Third, transcriptome-wide analysis following EF-2 knockdown would elucidate the precise molecular cascade leading to lethality, informing dosing strategies and identifying secondary vulnerabilities (9). Fourth, *in silico* molecular docking and virtual screening campaigns using the EF-2 structural model, benchmarked against the sordarin-eEF2 co-crystal structure precedent, could rapidly identify lead compounds for downstream biochemical and phenotypic validation (34). Fifth, the evaluation of EF-2 -targeted RNAi delivered via engineered symbiotic bacteria or nanoparticle platforms in semi-field and field cage settings represents a necessary pre-translational step. Paratransgenesis approaches using engineered bacterial symbionts have demonstrated substantial promise for *Plasmodium* inhibition, with some systems achieving over 97% parasite suppression, suggesting potential synergies with EF-2-directed interventions (34). Sixth, the study of EF-2’s evolutionary conservation and functional significance is necessary for other epidemiologically pertinent anopheline species. Through comparative sequence alignment, phylogenetic analysis, and structural modeling, we can evaluate the conservation of important amino acids and possible inhibitor-binding sites by using EF-2 orthologues from *An. gambiae*, *An. minimus*, *An. maculatus* and *An. kochi*. Comparative docking and molecular dynamics studies will determine whether species-specific amino acid substitutions can change ligand recognition or stability. This information will allow the prediction of the use of compounds targeting EF-2 in vector control interventions. Finally, integration of EF-2-targeting approaches within an Integrated Vector Management (IVM) framework, combining RNAi-based strategies with existing LLINs, IRS, and biological control, could extend the operational life of current tools while resistance continues to evolve (19).

A comparative dN/dS analysis shows a high level of conservation in the EF-2 across the entire genus Anopheles. Further studies are needed to incorporate other vectors of epidemiological importance that must be tested, especially members of the An. maculatus complex and An. kochi, and to include structural and molecular modeling analysis to test if there are differences in inhibitors binding and specificity because of species-specific amino acid substitutions.

## 8 Conclusion

Elongation Factor 2 is a novel molecular target for controlling *An. gambiae*. Elf2 (AGAP009441), a GTPase that occurs as a single copy and has no known backup system in *An. gambiae* qualifies as an ideal target because its inactivation is lethal. Hence, the leading role of Elongation Factor 2 is evident from the 61% knockdown efficiency achievable with the currently available dsRNA administration protocols. This means that mosquito survival can be significantly improved (p < 0.0001), and consequently, there is ample opportunity for improvement here. Three main challenges that need to be resolved regarding the targeting of elongation factor 2 include low knockdown efficiency, close sequence similarity to the human ortholog, and the need for efficient field development. Given the serious problem of growing insecticide resistance, which threatens the achievements of the last decades of malaria control, it is possible to conclude that elongation factor 2 provides researchers with an alternative means of mosquito control. In structural biology, delivery technologies, and ecological risk assessments will be necessary to achieve its full potential. EF-2 must be prioritized as a candidate for the next generation of malaria vector control.
